# The longitudinal effect of disseminating handwashing public health education to children in India via co-created, culturally relevant resources

**DOI:** 10.1099/acmi.0.000677.v3

**Published:** 2024-01-17

**Authors:** Sapphire Crosby, Sarah Younie, Harnesh Vijay Ujenia, Katie Laird

**Affiliations:** ^1^​ De Montfort University, Leicester, UK

**Keywords:** hand-hygiene, education, intervention, children, infection prevention, India

## Abstract

Infectious diseases are a leading cause of death for children from low- and middle-income countries (LMICs), often due to inadequate hand-hygiene. This study evaluates culturally relevant educational resources as a vehicle to disseminate the importance of handwashing amongst children in India. Employing a participatory action research (PAR) model and mixed methods, this follow-up longitudinal study evaluates a set of innovate educational handwashing resources and workshops specifically co-created for use in the State of Gujarat, and how they aid teachers in the teaching of hand-hygiene over a 3 year period. Working alongside local NGOs on-the-ground, teacher questionnaires (*n*=58) and focus groups including a brief questionnaire with teachers (*n*=35) were conducted to assess the impact of trainer workshops. In addition, pre- and post-workshop worksheets were conducted with children (*n*=98). Percentage change was calculated between children’s pre-and post-worksheet scores and a cumulative frequency of responses to each questionnaire criterion was measured. Data from the focus group found that the resources had been used in over 200 schools by more than 5000 children. In addition, 92.28 % of teachers said they would use the resources within their classrooms in India, with 58.16 % of pupils having an increased understanding of germs/handwashing directly after the workshop. Teachers reported that they are able to teach microbiology and handwashing more effectively. Furthermore, following a focus group, 100 % of teachers noted a reduction in childhood vomiting and diarrhoeal illnesses linked with insufficient hand-hygiene across 46 schools in the State of Gujarat since using the Germ’s Journey resources.

## Data Summary

The authors confirm all supporting data and protocols have been provided within the article or through supplementary data files, available in the online version of this article.

**Impact Statement**

Developing educational resources in low- and middle-income countries (LMICs), where preventable infectious diseases are the leading cause of childhood deaths, is crucial in infection prevention and control. Findings from this study show that the culturally relevant, co-created educational resources have improved children’s understanding of microbial transmission and hand-hygiene and increased teachers’ confidence to teach about microbiology, within the Gujarat state of India. Teachers noted the importance of the resources’ culturally relevant illustrations in teaching and learning. Importantly, over a 3 year period, since using the resources teachers noted a reduction in childhood illnesses, such as diarrhoea and vomiting, across 46 schools in the State of Gujarat, India. Recommendations for developing other educational resources in LMICs include adopting a co-creation approach to ensure authenticity.

## Introduction

It has been reported that approximately 9 % of children under 5 years old die annually from diarrhoea, often a symptom of present infection [1]. Therefore, stringent infection prevention such as comprehensive handwashing education and practices should facilitate an improvement in the number of children suffering from diarrhoea. Consequently, Ejemot-Nwadiaro *et al*. [2] found that promoting handwashing at schools in high-income countries prevents approximately 30 % of diarrhoeal episodes and a similar prediction is made for LMICs.

‘A Germ’s Journey’ is a set of interactive handwashing and infection-prevention resources, originally created by Microbiologist Katie Laird and Educationalist Sarah Younie in 2016. The resources were initially developed in the UK, to teach young children about microbial transmission and hand-hygiene due to the lack of such resources at the time [3]. Following in-depth scoping and international research trips, resources were also co-created with teachers, children and local specialists to be used globally, and were developed and disseminated across three continents (Africa, Asia and Europe), with a particular focus in low- and middle-income countries (LMICs) [4–6].

The resources comprise of UK, Gujarati and West Africa versions of a book, as well as webgames, handwashing songs/videos, posters and parent/teacher guides. All were co-created with experts and end-users to be culturally relevant and free at the point of access via the project’s website (www.germsjourney.com). In addition to being stand-alone resources, the resources are also used as part of a carousel-style children’s workshop. An initial pilot study [4], was conducted in 2017 using the UK version of the book as a scoping exercise to inform the development of new culturally relevant Gujarati resources. Then in 2019, a study [4] was conducted using the newly developed Gujarati resources, which found an increased understanding in 54 % of children when using the Gujarati book.

This paper outlines the findings of a follow-up study, 1 year after the implementation of the resources in schools (original study: [4]) in the State of Gujarat, India, focusing on the use of culturally relevant resources as a vehicle to disseminate the importance of handwashing amongst children and teachers, to address the morbidity/mortality associated with poor hand-hygiene.

To establish life-long handwashing habits that will continue into adulthood, children should be educated from a young age [2, 7]. Due to the typically egocentric (inability to understand a concept from anybody’s point of view other than one’s own) nature of children’s intellectual development during their early years [8], young children find it difficult to understand abstract concepts such as invisible microbes. When developing the resources, well-established pedagogic theories were adopted, namely, experiential learning [9, 10], which highlights the success of learning through hands-on experience, and zone of proximal development [11] and scaffolding [12], which emphasize the importance of parental/teacher involvement within the learning process [3, 4].

Developing resources for children, especially in LMICs where preventable diseases are a leading cause of childhood death [1], is crucial in infection prevention and control. Simply put, handwashing education, particularly within areas of socio-economic disadvantage, is lifesaving and now is even more pertinent due to the COVID-19 pandemic.

## Research questions

1. Does the Gujarati ‘A Germ’s Journey’ book influence children in India’s understanding of hand-hygiene and germ transmission?
2. Does the Gujarati ‘A Germ’s Journey’ book enable teachers to teach principles of hand-hygiene and germ transmission more effectively after being in use for 1 year?
3. Do the Germ’s Journey resources have an impact on reducing childhood illness in the State of Gujarat, India since first being used 1 year previously?

## Methods

### Developing effective culturally relevant Gujarati resources

After initially conducting workshops in 2017 using the UK resources in Ahmedabad, India, the resources were redeveloped so that children and teachers could fully engage with them. Working alongside non-governmental organizations (NGOs) (Table 1) within the Ahmedabad area, the resources were adapted to be culturally relevant and fit-for-purpose for teaching and learning within India. During the second visit to Ahmedabad in 2019, immediately after using the newly developed Gujarati book, 54 % of children had an increased understanding of germ transmission and handwashing, with teachers reporting positively following a trainer workshop [4]. The researchers worked alongside Gujarati translators during all workshops and all data collection materials were translated.

**Table 1.** NGO profiles

NGO Name	Profile
Manav Sadhna (MS)	Founded in 1995 ‘Strengthens underprivileged communities in Ahmedabad through programmes [including] Holistic Education, Nutrition, Health and Hygiene, Youth and Women’s Empowerment, Senior Care’[21]
Environmental Sanitation Institute (ESI)	Founded in 1985 Works ‘in the areas of training, construction, and supervision of environmental sanitation campaigns across India’ [22]
Together in Development and Education (TIDE)	Founded in 2014 ‘Aims to bring about a sustainable change in the educational system and improve access to quality education’ [23]

Whilst there is an undisputed need for culturally relevant public health resources and interventions, there is a lack of follow-up studies closely analysing the impact that such resources have. This follow-up study targets this lack of literature, to examine if such resources result in long-term societal benefits. In this case, the impact that the educational resources have had on children’s understanding, the teachers’ opportunity to teach the topic area more effectively and a decrease in the transmission of infectious diseases.

### Subjects and methods

Using models of participatory action research (PAR) and co-creation [4, 5], the researchers worked closely alongside NGOs, translators, end-users and stakeholders to develop the resources and conduct educational workshops. A mixed methods approach was used to collect data. This allowed the researchers to both quantify the teacher’s questionnaire responses and children’s worksheet data, whilst also gaining insightful information from teachers in the focus groups, enabling richer findings.

### Participants

Participants included 98 children aged between approximately 3–10 years who completed a pre-workshop and post-workshop worksheet. The workshop used the resources, including the Gujarati ‘A Germ’s Journey’ book.

The researchers also worked alongside Anganwadi (community school) teachers to either:

1. Run teacher/facilitator sessions where the teachers were shown how to effectively use the resources to aid hand-hygiene lessons with children (58 teachers) or
2. Assess how the teachers, that were trained in January 2019 by A Germ’s Journey, used the resources within their classrooms, and how the resources have impacted their confidence and supported them to teach about handwashing (35 teachers).

### Data collection tools

#### Teacher questionnaire

The questionnaire was completed by 58 teachers/facilitators across the three NGO institutes who had no previous experience of using the Gujarati resources. A trainer workshop was conducted with the teachers and included the researchers showing the Gujarati ‘A Germ’s Journey’ resources and the teachers observing the pupils partaking in their workshop. The questionnaire was completed by teachers after the workshop to determine the effectiveness of the resources. The questionnaire also investigated if the teachers felt more supported to undertake handwashing sessions in their own classrooms.

#### Children’s worksheet

The worksheet was completed by 98 children (aged 3–10 years) across two private schools in Ahmedabad, India that provided education for economically disadvantaged families (i.e. schools that charged minimal fees). These children had no previous exposure to the Gujarati resources. Initially, the pupils’ awareness about the topic of hand-hygiene was assessed using the pre-workshop worksheet. Next, the children participated in a carousel-style workshop. At the end of this workshop, an identical worksheet (post-workshop questionnaire) was used to assess how the workshop resources impacted their understanding.

#### Focus group

The focus group was conducted with 35 teachers in Ahmedabad, India who had previously been trained in how to use ‘A Germ’s Journey’ resources. A brief yes/no questionnaire was used to stimulate discussion, to determine if and how the teachers had been using the Gujarati resources in the previous 13 months, and how this has impacted their pupils’ awareness of handwashing. This allowed the researchers to assess how the resources had a longer-term impact on the teachers’ ability to teach pupils about hand-hygiene more effectively, and the teachers’ opinions on the quality of the resources. It also helped the researchers determine the societal impact and the reach of the resources. The session was undertaken and translated by a Gujarati speaker. The brief questionnaire was anonymous, and the researchers were not present whilst they were completed.

#### Ethics

The study received ethical approval from De Montfort University’s Health and Life Sciences Research Ethics Committee. The study adhered to the British Education Research Association ethical code of practice [13].

## Findings

To answer the research questions, data was obtained using a questionnaire for teachers about their current microbiology teaching practices (Table 2) and their views on the Germ’s Journey resources (Table 3). Children’s pre- and post-workshop questionnaires/worksheets were completed to measure their understanding directly before and after the workshop, with children’s average pre-workshop questionnaire test scores being calculated to gauge their pre-existing knowledge (Table 4). Focus groups and questionnaires were completed by teachers who had been using the Germ’s Journey resources to assess the long-term impact (Table 5).

**Table 2.** Teachers' questionnaire responses regarding previous teaching of microbiology/handwashing before using Germ’s Journey resources

**Statement**		**Percentage of teachers (%)**	
	**Yes**	**No**	**Unanswered**
Do you teach your pupils about microbiology?	74.14	22.41	3.45
Do you teach your pupils about handwashing?	91.38	0	8.62
Do you use handwashing resources with your pupils?	82.76	6.9	10.34

**Table 3.** Teachers' questionnaire responses following the workshop

**Statement**		**Percentage of teachers (%)**	
	**Agree**	**Strongly Agree**	**Unanswered**
The workshop has increased my confidence to teach microbiology/handwashing	31	69	0
The tips at the back of the book are useful to scaffold the children’s learning	32.76	63.79	3.45
The range of activities are useful to reinforce the children’s learning	29.31	68.97	1.72

**Table 4.** Children’s average pre-workshop test marks

Average pre-workshop test mark of pupils whose scores increased in the post-workshop test	13.70 out of 20
Average pre-workshop test mark of pupils whose scores decreased in the post-workshop test	16.04 out of 20
Average mark of the pupils that maintained the same mark in the pre- and post-workshop tests	16.05 out of 20

**Table 5.** Teachers' responses to 'A Germ’s Journey' follow-up questionnaire

Question	Answer
Did you teach health hygiene/germs/handwashing before having the ‘A Germ’s Journey’ resources?	Yes: 100 %
By having the ‘A Germ’s Journey’ resources do you now teach health hygiene/germs/handwashing more?	Yes: 100 %
Have the ‘A Germ’s Journey’ resources changed how you teach about health hygiene/germs/handwashing?	Yes: 100 %
Do you think that your children have a better understanding of health hygiene/germs/handwashing since you started using ‘A Germ’s Journey’ resources?	Yes: 100 %
Since learning about health hygiene/germs/handwashing using the ‘A Germ’s Journey’ resources, have your pupils changed their handwashing behaviour? (E.g., Do they wash their hands more?)	Yes: 100 %
Since using the ‘A Germ’s Journey’ resources have you seen a reduction in illness associated with vomiting and diarrhoea?	Yes: 100 %
**Additional comments:**	
The picture of a child vomiting in the book has stuck in the children’s minds and they recall it. The contents of the book have improved handwashing practices in children as the children feel they will get immediately sick if they don’t wash their hands. Children are now washing their hands as soon as they come into school. Before having the resources, the teachers would explain the concept of germs to the children but weren’t able to depict them. Now with the aid of the book, they are able to do so and thus children are able to understand more. Children still recall the glo-gel activity, even 13 months after it was conducted. Children have increased their frequency of handwashing as well as an improvement in their handwashing technique has been observed. Children are going home and teaching their parents/siblings about the advantages of handwashing. The pictures (in the Gujarati book) have helped the children visualize germs and helps to maintain the children’s attention.	

N.B - See Appendix D for a copy of the questionnaire.

### Teacher questionnaire

It was important for the researchers to learn how the teachers (*n*=58) educate pupils in microbiology principles and handwashing (Table 2). Whilst 91.38 % of the teachers reported that they do teach pupils about handwashing, only 74.14 % teach them about microbiology (i.e., microbial transfer and the consequences of inadequate handwashing practice). Over 6 % of the teachers do not use resources when teaching about handwashing. (See Appendix A for a copy of the questionnaire).

As part of the same questionnaire, the teachers (*n*=58) were presented with a series of statements regarding the Germ’s Journey resources used in the trainer workshop (Table 3). Overall, the findings show that 69 % of teachers (*n*=58) strongly agreed that participating in the trainer workshop increased their confidence to teach their pupils about handwashing and principles of microbiology. For statements 2 and 3 (Table 3), >60 % strongly agreed that the tips at the back of the book are helpful in scaffolding the children’s learning, and that the range of activities (made possible by the culturally relevant resources produced) are useful to reinforce children’s learning. Additionally, after completing the trainer workshop, 98.28 % stated that they would use the workshop to teach handwashing in their classrooms. (See Appendix A for a copy of the questionnaire).

### Children’s worksheet

Worksheets completed by the children (*n*=98) showed that 58.16 % had an increased understanding of germs/handwashing directly after participating in the workshop. The researchers assessed this by comparing each student’s pre- and post-workshop worksheet scores. A further 18.37 % maintained their scores, suggesting they neither increased nor decreased in their understanding.

Average pre-workshop questionnaire test scores were calculated (Table 4) for pupils of all three categories (those that gained understanding, those that displayed a loss of understanding, and those that maintained their level of understanding) to assess the pupils’ baseline knowledge. Worksheet scores were calculated based on whether children’s answers were correct (one mark) or incorrect (0 marks) out of a possible 20 marks (see Appendix B for the children’s worksheet and C for the answer/marking sheet).

### Focus groups

Findings from the focus groups and questionnaire with teachers (*n*=35) were used to assess the long-term impact of the Gujarati Germ’s Journey resources on (a) the teachers’ approach to and frequency of teaching about handwashing, (b) pupils’ awareness about handwashing and (c) the greater societal impact of these resources (see Appendix D for the questionnaire). Resources had been used in over 200 schools by more than 5000 children, with 100 % of teachers responding positively to all questions (Table 5).

Having presented the findings of the study according to the data collection method, the paper next discusses these findings in relation to the research questions.

## Discussion

**1. Does the Gujarati ‘A Germ’s Journey’ book influence children in India’s understanding of hand-hygiene and germ transmission?**

Results from the worksheet found that 58.16 % of children had an increase in their post-workshop score, suggesting that the use of culturally relevant resources has removed a cultural and language barrier for the children, making the information more accessible. An important issue with research showing an increase in learning development when using children’s native language and representing their native culture within classrooms [14–16]. Data from the previous study showed slightly lower results, with 54 % of children demonstrating an increased understanding following the workshops [4]. It is important to note that the children in the previous study were from areas of severe deprivation, making their access to frequent formal education limited, whereas the children in this study were enrolled at fee-paying schools.

Whilst handwashing knowledge increased for a large proportion of the children, almost 25 % of the children showed a reduction in their post-workshop score. One likely explanation for this is that for 45 of the children, the workshop was conducted an hour before the end of the school day, leaving limited time to complete the post-workshop worksheet, where children were visibly restless and keen to go home. Furthermore, the workshop is designed to be a fun, interactive experience [10], whereas completing a worksheet is less stimulating in comparison. Interestingly, the pupils who did improve their scores post-workshop had a lower average pre-workshop score (13.70) in comparison to those that either maintained (16.05) or had a lower score (16.04) (Table 4). Pupils that showed an increase in their knowledge were actually those that demonstrated the least understanding initially. This shows that the resources do influence children’s understanding, with a marked increase particularly with pupils who had lower baseline knowledge. The next research question investigates the resources’ impact on teachers.

**2. Does the Gujarati ‘A Germ’s Journey’ book enable teachers to teach principles of hand-hygiene and germ transmission more effectively after being in use for 1 year?**

Every teacher stated that having access to the Gujarati Germ’s Journey resources changed the way they teach their pupils about health-hygiene/germs/handwashing. In the focus group, multiple teachers commented on the culturally relevant illustrations in the book, explaining that before they had access to it, they would explain the concept of microbes but were unable to depict them (an important element when educating children about abstract concepts such as this) [8, 10–12]. However, with the book, they are now able to do so, and the children are better able to understand the concept of microbes and consequently the need to wash hands. The teachers recalled the illustration of the child vomiting in the book, commenting that their pupils better understand the consequences of not washing their hands, again highlighting that the illustrations, particularly, have improved the children’s understanding of hand-hygiene and aid teachers to educate pupils on its importance. This is further supported by Ouimet [17] who identified that having culturally relevant resources with authentic images will interest and relate to the pupils more, with Adukia *et al*. [18] noting the importance of cultural representation in children’s books. Additionally, the teachers stated that since using the Germ’s Journey resources, their pupils disseminate their knowledge to the wider community, by discussing the topic with their families, exemplifying how public health messages can be disseminated to all (including older) generations using school-based interventions (Table 5).

Teachers’ feedback across the 3 year study period has been consistently positive. When comparing these results to the previous study’s [4], in which the UK resources were used, the data has shown an increase in teachers’ views about the usefulness of the workshop (98.28 % when using the Gujarati book as opposed to 88–90 % when using the UK book), unsurprisingly demonstrating that teachers have found the co-created culturally relevant resources more useful and beneficial [17].

Furthermore, 100 % of the teachers stated that since using the Germ’s Journey resources, their pupils have changed their handwashing behaviour (Table 5), commenting that their pupils had increased their frequency of handwashing, and observing an improvement in their technique, highlighting the importance of interventions to increase knowledge, consequently leading to behaviour change [5]. Finally, this study considers the effect of the resources in relation to pupil illness.

**3. Do the Germ’s Journey resources have an impact on reducing childhood illness in the State of Gujarat, India since first being used 1 year previously?**

Perhaps most significantly, data found that since using the resources, 100 % of the teachers noticed a reduction in illness associated with vomiting and diarrhoea (Table 5). This reinforces studies such as Wilmot *et al*.’s [19], and Hoyle *et al.*’s [20] systematic review, which found that ‘school-based interventions significantly reduced respiratory tract and gastrointestinal infection-related absence’. When these results (Table 5) are further extrapolated across the 46 schools worked with, and the almost 1000 pupils that have been part of this journey, the results are even more significant and the wider societal impact of this study is apparent.

## Conclusion

Working alongside teachers and pupils in India, this longitudinal study sought to evaluate the impact of using co-created culturally relevant Germ’s Journey resources to effectively disseminate hand-hygiene education for children in the State of Gujarat, India over a 3 year period.

Findings demonstrate that using the Germ’s Journey resources has directly improved children’s understanding of the topic, has enabled teachers to effectively teach their pupils about germs and hand-hygiene and, arguably most significantly, has shown a reduction in illnesses associated with poor hand-hygiene across 46 schools in India. These results are further amplified as these resources have been used in approximately 200 schools, impacting the handwashing practices of over 5000 children. This not only indicates that the resources are effective in their aim for the participants in this study, but more significantly, highlight the potential that such resources and educational interventions can have more widely on society, particularly within LMICs. Although used within the context of a school settings, the positive findings suggest that this workshop intervention/resources could be used within hospitals and community health clinics/settings, as well as within other community groups to improve hand-hygiene understanding and practice, and reduce illness.

## Supplementary Data

Supplementary material 1
