# Supplementary material for: The longitudinal effect of disseminating handwashing public health education to children in India via co-created, culturally relevant resources
Source: Access Microbiol. 2024 Jan 17;6(1):000677.v3. doi: 10.1099/acmi.0.000677.v3 (PMC10866036; doi:10.1099/acmi.0.000677.v3)
Supplement: Supplementary material 1 [file acmi-6-677.v3-s001.pdf]

Appendices

Appendix A: Questionnaire ‘A Germ’s Journey’ for teachers/facilitators

	Question	Likert scale - please circle
1	Pupils were focused and engaged in the workshop	Strongly disagree / disagree / don’t know / agree / strongly agree
2	The workshop is useful as a teacher/early years’ assistant	Strongly disagree / disagree / don’t know / agree / strongly agree
3	The workshop has increased my confidence to teach microbiology/ handwashing	Strongly disagree / disagree / don’t know / agree / strongly agree
4	Pupils were focused and engaged in the book	Strongly disagree / disagree / don’t know / agree / strongly agree
5	The tips at the back of the book are useful to scaffold the children’s learning	Strongly disagree / disagree / don’t know / agree / strongly agree
6	What activity did the pupils enjoy most about the workshop? Tick all that apply	1) The book reading activity<br>2) The website activity<br>3) The glo-gel activity<br>4) The colouring activity<br>5) Anything else
7	The range of activities are useful to reinforce the children’s learning	Strongly disagree / disagree / don’t know / agree / strongly agree
8	Can you see yourself using the workshop to teach handwashing in school?	Yes/no / other comments
9	Do you think the children’s handwashing practice will increase after the workshop?	Yes/no / other comments
10	What did you enjoy most/find most useful about the workshop?	
11	What would you change about the workshop?	
12	Any other comments on the workshop activities?	
13	Do you teach your pupils about microbiology and if so, what do you teach specifically?	Yes/ no<br>Comments:

14	Do you teach your pupils about handwashing and if so, what do you teach specifically?	Yes/ no<br>Comments:
15	Do you use handwashing resources with your pupils? If so, what resources do you use?	Yes/ no<br>Comments:

Appendix B: Children's worksheet/questionnaire

A (Pre-Workshop)
Total:

Name: ______________________ ~~Age:~~ ______

A (Pre-Workshop)

A1. When do you need to wash your hands?

Please circle either 'yes' or 'no'.

		✔	✘
1	After going to the toilet	Yes	No
2	When you wake up	Yes	No
3	After touching animals	Yes	No
4	After coughing or sneezing into your hands	Yes	No
5	After playing outside	Yes	No
6	After reading a book	Yes	No
7	Before eating	Yes	No

☐

A2. Do you know how to make germs go away?
Please circle your answer.

☹ No	😐 Not sure	☺ Yes

☐

A3. Do you know where germs live?
Please circle your answer.

☹ No	😐 Not sure	☺ Yes

☐

A4. What types of water are **safe** to use when you wash your hands? ✔

Please circle your answer.

Sewage Water	Flood Water	Tap Water

☐

Name: ______________________ ~~Age:~~ ______

A5. Please circle the places where you would find germs

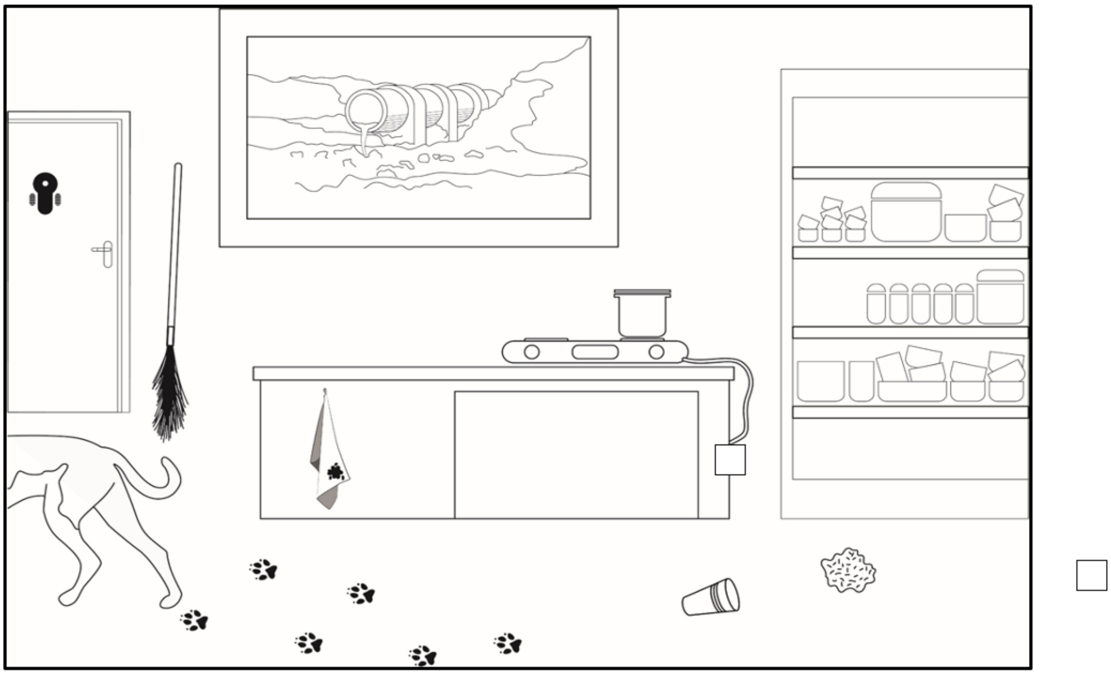

Appendix C: Children's worksheet/questionnaire answer/marking sheet

A (Pre-Workshop)
Total:

/20

Name: ______________________________ Age:_______

(વર્ગ શરૂ કરતા પહેલાં)

A1. આ વિકલ્પોમાંથી, તમારે ક્યારે હાથ ધોવાની જરૂર છે?

આ વિકલ્પોમાંથી, કૃપા કરીને 'હા' અથવા 'ના' વર્તુળ કરો.

		✔	✘
૧	ટોઇલેટનો ઉપયોગ કર્યા પછી	હા	ના
૨	જાગતી વખતે	હા	ના
૩	પ્રાણીઓ સ્પર્શ કરીયા પછી	હા	ના
૪	તમારા હાથમાં ખાંસી અથવા છીંક્યા પછી	હા	ના
૫	બહાર રમવા પછી	હા	ના
૬	એક પુસ્તક વાંચ્યા પછી	હા	ના
૭	ખાવા પહેલાં	હા	ના

7

A2. શું તમે જાણો છો કે જંતુઓ કેવી રીતે દૂર કરવામાં આવે છે?
કૃપા કરીને તમારા જવાબને વર્તુળ કરો.

☹ 0 ના

😐 1 મને ખબર નથી

☺ 2 હા

2

A3. શું તમે જાણો છો કે જંતુઓ ક્યાં રહે છે?
કૃપા કરીને તમારા જવાબને વર્તુળ કરો.

☹ 0 ના

😐 1 મને ખબર નથી

☺ 2 હા

2

A4.જ્યારે તમે હાથ ધોશો ત્યારે કયા પ્રકારનાં પાણીનો ઉપયોગ કરવો સલામત છે?

✔

કૃપા કરીને તમારા જવાબને વર્તુળ કરો.

ગટર નું પાણી	પૂર નું પાણી	નળ નું પાણી

1

Name: ______________________________ Age:______

A5. કૃપા કરીને આ ચિત્રમાં સ્થાનોને વર્તુળ કરો જ્યાં તમને જંતુઓ મળશે

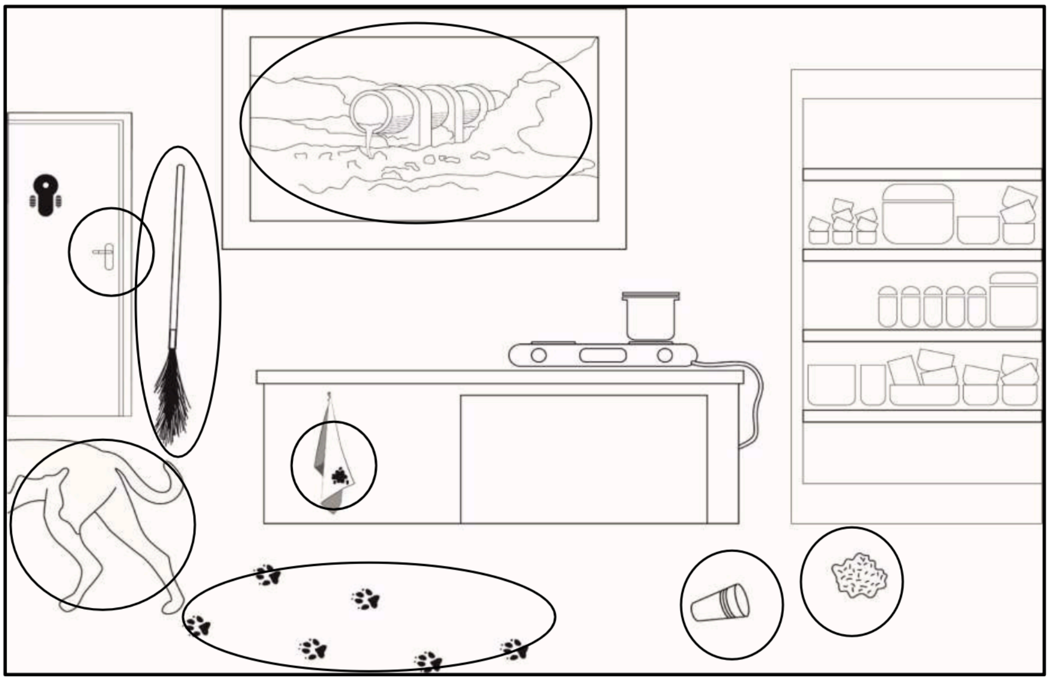

8

Appendix D: Germ's Journey Focus Group Post-Intervention Questionnaire for Teachers

1	Did you teach health hygiene/germs/handwashing before having the Germ's Journey resources?	Yes / No
2	By having the Germ's Journey resources do you now teach health hygiene/germs/handwashing more?	Yes / No
3	Have the Germ's Journey resources changed how you teach health hygiene/germs/handwashing?	Yes / No
4	Do you think that your children have a better understanding of health hygiene/germs/handwashing since you started using the Germ's Journey resources?	Yes / No
5	Since learning about health hygiene/germs/handwashing using the Germ's Journey resources, have your students changed their handwashing behaviour?<br><br>E.g , do they wash their hands more?	Yes / No
6.	Since using the Germ's Journey resources have you seen a reduction in illness associated with vomiting and diarrhoea?	Yes / No
